# Evaluating the pandemic’s impact on surgical site infections after abdominal surgery in Italy

**DOI:** 10.1093/eurpub/ckac130.016

**Published:** 2022-10-25

**Authors:** HSMA Elhadidy, G Paladini, E Ugliono, AR Cornio, V Bordino, C Vicentini, CM Zotti

**Affiliations:** 1 Department of Public Health Sciences, Università degli Studi di Torino, Turin, Italy; 2 Department of Surgical Sciences, Università degli Studi di Torino, Turin, Italy

## Abstract

**Background:**

The COVID-19 pandemic led to important disruptions in surgical activity. The aim of this study was to evaluate the impact of COVID-19 on abdominal surgery outcomes in the region of Piedmont, in northern Italy.

**Methods:**

Data were gathered from 42 hospitals participating in the regional surveillance network from 2018 to 2020. SSI, overall mortality and case fatality rates (CFR) were calculated, comparing 2020 to mean 2018-19 data. Chi-squared tests were used to assess both the differences among the proportion of urgent and oncological procedures (based on ICD-9-CM codes) and rates between the two periods. Subgroup analyses on 2020 data were carried out comparing urgent vs. elective and oncological vs. non-oncological procedures using chi-squared tests. Analyses were performed using SPSS v. 28.0.1.0.

**Results:**

5407 procedures were recorded in 2018-19; 310 SSIs and 120 deaths were observed. The mean proportions of urgent and oncological operations were, respectively, 21.90% and 43.24%. In 2020, 1057 procedures were recorded, along with 44 SSIs and 29 deaths. 34.44% of procedures were urgent and 39.74% oncological. The mean 2018-2019 SSI rate was 5.73%, with an overall mortality of 2.22% and a CFR of 7.42%. The SSI rate in 2020 was 4.16%, with an overall mortality of 2.74% and a CFR of 9.09%. The proportion of urgent procedures significantly differed between the two periods (p < 0.001), as did the proportion of oncological procedures and SSI rates (both p = 0.05). Considering 2020, significant differences were found comparing overall mortality between urgent vs. elective procedures (4.95% vs. 1.59%, p = 0.002) and comparing SSI rates between oncological vs. non-oncological patients (3.57% vs. 2.20%, p = 0.02).

**Conclusions:**

During the pandemic, patients undergoing surgical procedures significantly differed, reflecting public health decisions. Even though these differences did not reach statistical significance, overall mortality and CFR increased in 2020.

**Key messages:**

- Results of this study suggest the COVID-19 pandemic had a negative impact on outcomes after abdominal surgery.
- Further longer-term studies are needed to assess the impact of policies implemented during the pandemic, to help inform future pandemic plans.

